# 2-(5-Bromo­pent­yl)-4-chloro-5-[2-(4-meth­oxy­phen­yl)ethyl­amino]­pyridazin-3(2*H*)-one

**DOI:** 10.1107/S1600536810030102

**Published:** 2010-08-04

**Authors:** Hai-Quan Wang, Wang-Zhong Chen, Wen-Hua Chen, Bao-Min Xi

**Affiliations:** a School of Pharmaceutical Science, Southern Medical University, Guangzhou 510515, Guangdong, People’s Republic of China

## Abstract

The asymmetric unit of the title compound, C_18_H_23_BrClN_3_O_2_, consists of two mol­ecules which exhibit different conformations of the pentyl chains [C—C—C—C torsion angles of −60.4 (4) and 175.8 (3)°]. The crysal packing exhibits a chain structure, generated through the O atom of the pyridazinone forming a hydrogen bond with the N—H group of an adjacent mol­ecule.

## Related literature

The title compound is an inter­mediate in the synthesis of Alpha1-AR antagonists. For the biological applications of Alpha1-AR antagonists, see: Guderman *et al.* (1995 ▶); Cavalli *et al.* (1997 ▶); Pallavicini *et al.* (2006 ▶). For similar phenyl­piperazinepyridazinone derivatives synthesized as potential Alpha1-AR antagonists, see: Xi *et al.* (2006 ▶).
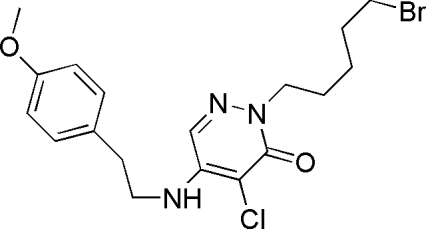


## Experimental

### 

#### Crystal data

C_18_H_23_BrClN_3_O_2_
*M* *_r_* = 428.75
Triclinic, 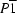

*a* = 9.7728 (14) Å
*b* = 12.6178 (19) Å
*c* = 15.500 (2) Å
α = 94.803 (2)°
β = 96.380 (2)°
γ = 91.035 (2)°
*V* = 1892.1 (5) Å^3^
*Z* = 4
Mo *K*α radiation
μ = 2.33 mm^−1^
*T* = 110 K
0.35 × 0.15 × 0.1 mm

#### Data collection

Rigaku Mercury diffractometer
Absorption correction: multi-scan (*REQAB*; Jacobson, 1998 ▶) *T*_min_ = 0.933, *T*_max_ = 0.975
15893 measured reflections
8131 independent reflections
6225 reflections with *I* > 2σ(*I*)
*R*_int_ = 0.036

#### Refinement

*R*[*F* ^2^ > 2σ(*F* ^2^)] = 0.044
*wR*(*F* ^2^) = 0.143
*S* = 0.99
8131 reflections
453 parameters
H-atom parameters constrained
Δρ_max_ = 1.39 e Å^−3^
Δρ_min_ = −0.88 e Å^−3^

### 

Data collection: *CrystalClear* (Rigaku/MSC, 2001 ▶); cell refinement: *CrystalClear*; data reduction: *CrystalStructure* (Rigaku/MSC, 2004 ▶); program(s) used to solve structure: *SHELXS97* (Sheldrick, 2008 ▶); program(s) used to refine structure: *SHELXL97* (Sheldrick, 2008 ▶); molecular graphics: *ORTEPII* (Johnson, 1976 ▶); software used to prepare material for publication: *SHELXL97*.

## Supplementary Material

Crystal structure: contains datablocks global, I. DOI: 10.1107/S1600536810030102/ez2210sup1.cif

Structure factors: contains datablocks I. DOI: 10.1107/S1600536810030102/ez2210Isup2.hkl

Additional supplementary materials:  crystallographic information; 3D view; checkCIF report

## Figures and Tables

**Table 1:** Hydrogen-bond geometry (Å, °)

*D*—H⋯*A*	*D*—H	H⋯*A*	*D*⋯*A*	*D*—H⋯*A*
N3—H3⋯O3^i^	0.88	2.11	2.796 (3)	135
N6—H6⋯O1^ii^	0.88	2.06	2.815 (3)	143

Symmetry codes: (i) 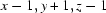; (ii) 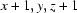.

**Fig. 1.**
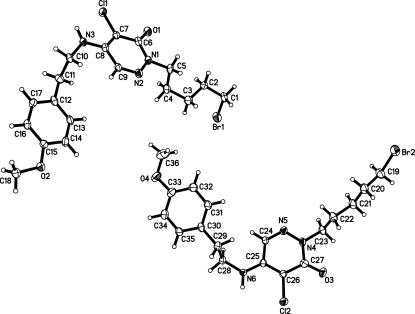
ORTEP-II (Johnson, 1976) plot of complex (I) at the 30% probability level. Hydrogen atoms are drawn as spheres of arbitrary radii.

**Fig. 2.**
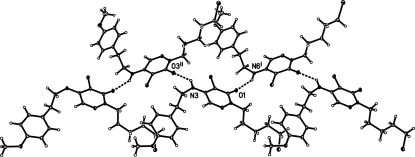
One-dimensional chain structure along the b axis formed via H-bonding interactions i: x - 1, y,z - 1 ii: x - 1,y + 1, z - 1

## supplementary crystallographic information

### Comment

Alpha1-adrenoceptors (Alpha1-AR) are members of the super family of seven transmembrane G protein coupled receptors (GPCR) (Guderman *et al.*, 1995) and regulate several important physiological processes. In recent years, the search for new alpha1-AR antagonists has intensified due to their therapeutic potential in the treatment of hypertension (Cavalli *et al.*, 1997) and benign prostatic hypertrophy (Pallavicini *et al.*, 2006). In the course of our studies on phenylpiperazinepyridazinone derivatives as potential Alpha1-AR antagonists, we have synthesized a range of compounds (Xi *et al.*, 2006) which show good blocking activities toward Alpha1-AR. Phenylpiperazinepyridazinone derivatives are a type of Alpha1-AR antagonist; the title compound is a key intermediate in the synthesis of phenylpiperazinepyridazinone derivatives.

The asymmetric unit of the title compound consists of two molecules which differ from one another crystallographically. The largest difference between the two molecules is in the conformations of the pentyl chains, indicated by the C2-C3-C4-C5 and C20-C21-C22-C23 torsion angles of -60.4 (4)° and 175.8 (3)°, respectively. The molecules contain pyridazinone and benzene rings, which are orientated at angles of 13.28 (17)° and 23.34 (14)° with respect to each other in the two molecules. The one dimensional chain structure found in the crystal packing is formed through intermolecular N—H···O hydrogen bonds.

### Experimental

A mixture of 5-(4-methoxyphenethylamino)-4-chloropyridazin-3(2*H*)-one (0.3 g), K_2_CO_3_ (0.22 g), 1,5-dibromopentane(0.34 g), and acetone (16 ml) were heated to reflux of the solvent for 10 h. After cooling, the resulting precipitate was filtered off and filtrate was evaporated. The residue was chromatographed on silica gel with petroleum-ethyl acetate (3:2 with TEA) as eluent to give the title compound (0.169 g, 36.7%). Crystals suitable for X-ray analysis were obtained from the slow evaporation of a chloroform solution (m.p. 355–356 K).

### Refinement

The final difference Fourier map had a peak of 1.40 e Å^-3^ at about 0.876 Å from Br1, H atoms were positioned geometrically and refined using the riding-model approximation, with C—H = 0.93 or 0.96 Å, O—H= 0.82 Å, N—H = 0.86 Å, and *U*_iso_(H) = 1.2*U*_eq_(C,*N*) or *U*_iso_(H) = 1.5*U*_eq_ (methyl C and O).

### Crystal data

**Table:**

C_18_H_23_BrClN_3_O_2_	*Z* = 4
*M**_r_* = 428.75	*F*(000) = 880
Triclinic, *P*1	*D*_x_ = 1.505 Mg m^−^^3^
Hall symbol: -p 1	Mo *K*α radiation, λ = 0.71073 Å
*a* = 9.7728 (14) Å	Cell parameters from 16062 reflections
*b* = 12.6178 (19) Å	θ = 3.0–27.1°
*c* = 15.500 (2) Å	µ = 2.33 mm^−^^1^
α = 94.803 (2)°	*T* = 110 K
β = 96.380 (2)°	Flake, colorless
γ = 91.035 (2)°	0.35 × 0.15 × 0.1 mm
*V* = 1892.1 (5) Å^3^	

### Data collection

**Table:**

Rigaku Mercury diffractometer	8131 independent reflections
Radiation source: fine-focus sealed tube	6225 reflections with *I* > 2σ(*I*)
graphite	*R*_int_ = 0.036
ω scans	θ_max_ = 27.1°, θ_min_ = 1.6°
Absorption correction: multi-scan (REQAB; Jacobson, 1998)	*h* = −12→12
*T*_min_ = 0.933, *T*_max_ = 0.975	*k* = −16→16
15893 measured reflections	*l* = −19→19

### Refinement

**Table:**

Refinement on *F*^2^	Primary atom site location: structure-invariant direct methods
Least-squares matrix: full	Secondary atom site location: difference Fourier map
*R*[*F*^2^ > 2σ(*F*^2^)] = 0.044	Hydrogen site location: inferred from neighbouring sites
*wR*(*F*^2^) = 0.143	H-atom parameters constrained
*S* = 0.99	*w* = 1/[σ^2^(*F*_o_^2^) + (0.1*P*)^2^] where *P* = (*F*_o_^2^ + 2*F*_c_^2^)/3
8131 reflections	(Δ/σ)_max_ = 0.001
453 parameters	Δρ_max_ = 1.39 e Å^−^^3^
0 restraints	Δρ_min_ = −0.88 e Å^−^^3^

### Special details

**Table:**

Geometry. All e.s.d.'s (except the e.s.d. in the dihedral angle between two l.s. planes) are estimated using the full covariance matrix. The cell e.s.d.'s are taken into account individually in the estimation of e.s.d.'s in distances, angles and torsion angles; correlations between e.s.d.'s in cell parameters are only used when they are defined by crystal symmetry. An approximate (isotropic) treatment of cell e.s.d.'s is used for estimating e.s.d.'s involving l.s. planes.
Refinement. Refinement of *F*^2^ against ALL reflections. The weighted *R*-factor *wR* and goodness of fit *S* are based on *F*^2^, conventional *R*-factors *R* are based on *F*, with *F* set to zero for negative *F*^2^. The threshold expression of *F*^2^ > σ(*F*^2^) is used only for calculating *R*-factors(gt) *etc*. and is not relevant to the choice of reflections for refinement. *R*-factors based on *F*^2^ are statistically about twice as large as those based on *F*, and *R*- factors based on ALL data will be even larger.

### Fractional atomic coordinates and isotropic or equivalent isotropic displacement parameters (Å^2^)

**Table:**

	*x*	*y*	*z*	*U*_iso_*/*U*_eq_	
Br1	0.30298 (4)	0.50393 (3)	0.45799 (2)	0.02925 (12)	
Cl1	0.17837 (8)	0.99896 (6)	−0.02591 (5)	0.02143 (18)	
O1	0.2247 (2)	0.80242 (16)	0.06417 (15)	0.0224 (5)	
O2	0.9083 (2)	1.48186 (17)	0.40044 (15)	0.0226 (5)	
C8	0.4160 (3)	1.0519 (2)	0.0756 (2)	0.0163 (6)	
C12	0.5870 (3)	1.3402 (2)	0.2271 (2)	0.0177 (6)	
N3	0.4189 (3)	1.14797 (19)	0.04352 (18)	0.0184 (5)	
H3	0.3616	1.1592	−0.0026	0.022*	
N2	0.5283 (3)	0.9306 (2)	0.17251 (18)	0.0200 (6)	
N1	0.4273 (3)	0.85810 (19)	0.14458 (17)	0.0185 (6)	
C6	0.3153 (3)	0.8731 (2)	0.0839 (2)	0.0179 (6)	
C1	0.3895 (4)	0.4850 (2)	0.3507 (2)	0.0221 (7)	
H1A	0.4903	0.4804	0.3651	0.027*	
H1B	0.3552	0.4173	0.3177	0.027*	
C15	0.8034 (3)	1.4410 (2)	0.3407 (2)	0.0183 (6)	
C9	0.5230 (3)	1.0221 (2)	0.1399 (2)	0.0183 (6)	
H9	0.5947	1.0730	0.1599	0.022*	
C17	0.6400 (3)	1.4397 (2)	0.2130 (2)	0.0182 (6)	
H17	0.6026	1.4736	0.1636	0.022*	
C10	0.5136 (3)	1.2340 (2)	0.0825 (2)	0.0195 (6)	
H10A	0.5181	1.2886	0.0406	0.023*	
H10B	0.6069	1.2055	0.0941	0.023*	
C16	0.7462 (3)	1.4910 (2)	0.2694 (2)	0.0181 (6)	
H16	0.7791	1.5596	0.2591	0.022*	
C7	0.3153 (3)	0.9743 (2)	0.0495 (2)	0.0167 (6)	
C14	0.7528 (3)	1.3408 (2)	0.3553 (2)	0.0214 (7)	
H14	0.7913	1.3063	0.4042	0.026*	
C4	0.3957 (4)	0.7748 (3)	0.2796 (2)	0.0288 (8)	
H4A	0.2954	0.7865	0.2757	0.035*	
H4B	0.4434	0.8396	0.3102	0.035*	
C13	0.6468 (3)	1.2917 (2)	0.2989 (2)	0.0210 (7)	
H13	0.6140	1.2231	0.3093	0.025*	
C2	0.3588 (3)	0.5764 (2)	0.2945 (2)	0.0219 (7)	
H2A	0.3890	0.5579	0.2363	0.026*	
H2B	0.2581	0.5862	0.2864	0.026*	
C3	0.4294 (4)	0.6802 (3)	0.3332 (2)	0.0296 (8)	
H3A	0.5303	0.6708	0.3391	0.035*	
H3B	0.4023	0.6967	0.3924	0.035*	
C11	0.4702 (3)	1.2862 (2)	0.1676 (2)	0.0208 (7)	
H11A	0.3995	1.3392	0.1536	0.025*	
H11B	0.4274	1.2311	0.1987	0.025*	
C18	0.9452 (4)	1.5921 (2)	0.4003 (2)	0.0251 (7)	
H18A	0.9844	1.6039	0.3463	0.038*	
H18B	1.0135	1.6131	0.4503	0.038*	
H18C	0.8630	1.6348	0.4043	0.038*	
C5	0.4380 (4)	0.7591 (2)	0.1882 (2)	0.0225 (7)	
H5A	0.5341	0.7350	0.1912	0.027*	
H5B	0.3781	0.7031	0.1538	0.027*	
Br2	1.17053 (4)	−0.11846 (3)	0.48534 (2)	0.03305 (12)	
Cl2	1.32193 (8)	0.51689 (6)	1.02756 (5)	0.02196 (18)	
O3	1.2740 (2)	0.29549 (18)	0.94660 (16)	0.0257 (5)	
N6	1.0840 (3)	0.64087 (19)	0.95016 (17)	0.0178 (5)	
H6	1.1411	0.6650	0.9957	0.021*	
N4	1.0735 (3)	0.32485 (19)	0.86383 (18)	0.0185 (6)	
N5	0.9745 (3)	0.3881 (2)	0.83069 (18)	0.0197 (6)	
O4	0.6028 (2)	0.9151 (2)	0.60480 (17)	0.0309 (6)	
C35	0.8609 (3)	0.8865 (2)	0.7875 (2)	0.0195 (6)	
H35	0.8914	0.9247	0.8419	0.023*	
C26	1.1866 (3)	0.4692 (2)	0.9527 (2)	0.0162 (6)	
C25	1.0874 (3)	0.5372 (2)	0.9223 (2)	0.0161 (6)	
C27	1.1857 (3)	0.3584 (2)	0.9229 (2)	0.0193 (6)	
C20	1.1627 (4)	0.0353 (3)	0.6350 (2)	0.0250 (7)	
H20A	1.1397	0.0879	0.5920	0.030*	
H20B	1.2642	0.0338	0.6471	0.030*	
C24	0.9815 (3)	0.4878 (2)	0.8582 (2)	0.0173 (6)	
H24	0.9117	0.5320	0.8346	0.021*	
C34	0.7561 (3)	0.9263 (3)	0.7332 (2)	0.0228 (7)	
H34	0.7160	0.9917	0.7502	0.027*	
C28	0.9910 (3)	0.7150 (2)	0.9092 (2)	0.0184 (6)	
H28A	0.9869	0.7797	0.9495	0.022*	
H28B	0.8973	0.6821	0.8980	0.022*	
C32	0.7701 (4)	0.7760 (3)	0.6286 (2)	0.0252 (7)	
H32	0.7400	0.7382	0.5740	0.030*	
C31	0.8749 (4)	0.7375 (2)	0.6842 (2)	0.0230 (7)	
H31	0.9154	0.6723	0.6672	0.028*	
C29	1.0361 (3)	0.7468 (2)	0.8230 (2)	0.0206 (7)	
H29A	1.1123	0.8005	0.8360	0.025*	
H29B	1.0721	0.6835	0.7917	0.025*	
C30	0.9227 (3)	0.7915 (2)	0.7640 (2)	0.0188 (6)	
C33	0.7092 (3)	0.8711 (3)	0.6540 (2)	0.0221 (7)	
C23	1.0566 (3)	0.2119 (2)	0.8319 (2)	0.0213 (7)	
H23A	0.9571	0.1942	0.8172	0.026*	
H23B	1.0922	0.1681	0.8791	0.026*	
C22	1.1304 (3)	0.1835 (2)	0.7524 (2)	0.0230 (7)	
H22A	1.2309	0.1954	0.7679	0.028*	
H22B	1.0997	0.2302	0.7062	0.028*	
C21	1.1006 (4)	0.0676 (2)	0.7185 (2)	0.0239 (7)	
H21A	1.1370	0.0215	0.7639	0.029*	
H21B	0.9996	0.0551	0.7081	0.029*	
C36	0.5565 (4)	0.8604 (4)	0.5224 (3)	0.0427 (10)	
H36A	0.6336	0.8535	0.4871	0.064*	
H36B	0.4835	0.9006	0.4924	0.064*	
H36C	0.5205	0.7895	0.5309	0.064*	
C19	1.1062 (4)	−0.0739 (3)	0.5981 (2)	0.0277 (8)	
H19A	1.0043	−0.0731	0.5909	0.033*	
H19B	1.1346	−0.1266	0.6400	0.033*	

### Atomic displacement parameters (Å^2^)

**Table:**

	*U*^11^	*U*^22^	*U*^33^	*U*^12^	*U*^13^	*U*^23^
Br1	0.0390 (2)	0.02394 (19)	0.0274 (2)	0.00207 (15)	0.01438 (16)	0.00288 (14)
Cl1	0.0201 (4)	0.0170 (4)	0.0258 (4)	−0.0022 (3)	−0.0048 (3)	0.0038 (3)
O1	0.0244 (12)	0.0140 (10)	0.0280 (13)	−0.0066 (9)	0.0003 (10)	0.0017 (9)
O2	0.0269 (12)	0.0156 (11)	0.0238 (12)	−0.0014 (9)	−0.0033 (10)	0.0020 (9)
C8	0.0176 (15)	0.0124 (13)	0.0188 (16)	−0.0003 (11)	0.0027 (12)	0.0010 (11)
C12	0.0166 (15)	0.0138 (14)	0.0234 (17)	0.0001 (12)	0.0074 (12)	−0.0008 (12)
N3	0.0205 (13)	0.0117 (12)	0.0216 (14)	−0.0044 (10)	−0.0034 (11)	0.0018 (10)
N2	0.0210 (14)	0.0133 (12)	0.0248 (15)	−0.0009 (10)	−0.0001 (11)	0.0011 (11)
N1	0.0232 (14)	0.0107 (12)	0.0211 (14)	−0.0032 (10)	−0.0001 (11)	0.0032 (10)
C6	0.0190 (15)	0.0123 (14)	0.0232 (17)	0.0008 (12)	0.0063 (12)	0.0004 (12)
C1	0.0311 (18)	0.0130 (14)	0.0247 (18)	0.0022 (13)	0.0132 (14)	0.0020 (12)
C15	0.0219 (16)	0.0167 (14)	0.0163 (16)	0.0030 (12)	0.0029 (12)	−0.0006 (12)
C9	0.0207 (15)	0.0109 (13)	0.0224 (17)	−0.0009 (12)	−0.0002 (13)	0.0004 (12)
C17	0.0198 (15)	0.0161 (14)	0.0191 (16)	0.0008 (12)	0.0018 (12)	0.0049 (12)
C10	0.0235 (16)	0.0107 (13)	0.0234 (17)	−0.0046 (12)	0.0007 (13)	0.0000 (12)
C16	0.0203 (15)	0.0131 (14)	0.0219 (17)	−0.0002 (12)	0.0056 (12)	0.0032 (12)
C7	0.0178 (15)	0.0138 (14)	0.0183 (16)	0.0003 (12)	0.0013 (12)	0.0013 (12)
C14	0.0259 (17)	0.0153 (15)	0.0237 (17)	0.0067 (13)	0.0033 (13)	0.0043 (13)
C4	0.047 (2)	0.0127 (15)	0.0263 (19)	0.0006 (15)	0.0004 (16)	0.0043 (13)
C13	0.0306 (18)	0.0107 (13)	0.0219 (17)	−0.0010 (12)	0.0055 (13)	0.0004 (12)
C2	0.0279 (17)	0.0167 (15)	0.0203 (17)	0.0012 (13)	−0.0022 (13)	0.0029 (12)
C3	0.045 (2)	0.0172 (16)	0.0253 (19)	−0.0039 (15)	−0.0041 (16)	0.0031 (14)
C11	0.0222 (16)	0.0177 (15)	0.0220 (17)	−0.0038 (12)	0.0031 (13)	−0.0015 (13)
C18	0.0314 (18)	0.0172 (15)	0.0258 (18)	−0.0032 (13)	−0.0012 (14)	0.0032 (13)
C5	0.0297 (18)	0.0112 (14)	0.0270 (18)	−0.0007 (12)	0.0016 (14)	0.0055 (12)
Br2	0.0400 (2)	0.0281 (2)	0.0312 (2)	0.00742 (16)	0.00531 (16)	0.00060 (15)
Cl2	0.0204 (4)	0.0195 (4)	0.0244 (4)	−0.0012 (3)	−0.0046 (3)	0.0026 (3)
O3	0.0289 (13)	0.0178 (11)	0.0300 (14)	0.0055 (10)	−0.0025 (10)	0.0075 (10)
N6	0.0200 (13)	0.0139 (12)	0.0179 (14)	−0.0004 (10)	−0.0042 (10)	0.0011 (10)
N4	0.0208 (13)	0.0114 (12)	0.0233 (15)	0.0018 (10)	0.0018 (11)	0.0027 (10)
N5	0.0212 (14)	0.0158 (12)	0.0221 (14)	0.0003 (10)	0.0000 (11)	0.0051 (11)
O4	0.0274 (13)	0.0332 (14)	0.0315 (14)	0.0042 (11)	−0.0053 (11)	0.0107 (11)
C35	0.0220 (16)	0.0149 (14)	0.0215 (17)	−0.0038 (12)	0.0020 (13)	0.0023 (12)
C26	0.0169 (15)	0.0146 (14)	0.0172 (15)	−0.0004 (11)	0.0014 (12)	0.0026 (12)
C25	0.0165 (15)	0.0132 (14)	0.0193 (16)	0.0000 (11)	0.0030 (12)	0.0029 (12)
C27	0.0249 (17)	0.0180 (15)	0.0156 (16)	−0.0024 (13)	0.0038 (13)	0.0047 (12)
C20	0.0243 (17)	0.0181 (16)	0.032 (2)	0.0003 (13)	0.0035 (14)	0.0002 (14)
C24	0.0201 (15)	0.0121 (13)	0.0195 (16)	0.0002 (11)	0.0006 (12)	0.0020 (12)
C34	0.0232 (17)	0.0175 (15)	0.0289 (19)	0.0015 (13)	0.0056 (14)	0.0052 (13)
C28	0.0208 (16)	0.0140 (14)	0.0207 (17)	0.0022 (12)	0.0020 (13)	0.0034 (12)
C32	0.0319 (19)	0.0201 (16)	0.0229 (18)	−0.0042 (14)	−0.0013 (14)	0.0042 (13)
C31	0.0310 (18)	0.0143 (14)	0.0237 (17)	−0.0011 (13)	0.0025 (14)	0.0020 (13)
C29	0.0225 (16)	0.0173 (15)	0.0229 (17)	0.0015 (12)	0.0032 (13)	0.0051 (12)
C30	0.0222 (16)	0.0121 (14)	0.0230 (17)	−0.0017 (12)	0.0060 (13)	0.0027 (12)
C33	0.0184 (16)	0.0223 (16)	0.0262 (18)	−0.0035 (13)	−0.0010 (13)	0.0103 (13)
C23	0.0265 (17)	0.0109 (14)	0.0262 (18)	−0.0031 (12)	0.0015 (14)	0.0028 (12)
C22	0.0264 (17)	0.0132 (14)	0.0300 (19)	−0.0048 (13)	0.0068 (14)	0.0020 (13)
C21	0.0301 (18)	0.0122 (14)	0.0293 (19)	−0.0029 (13)	0.0032 (14)	0.0021 (13)
C36	0.033 (2)	0.055 (3)	0.038 (2)	−0.0014 (19)	−0.0101 (18)	0.008 (2)
C19	0.038 (2)	0.0203 (16)	0.0257 (19)	−0.0001 (14)	0.0077 (15)	0.0003 (14)

### Geometric parameters (Å, °)

**Table:**

Br1—C1	1.947 (3)	Br2—C19	1.964 (3)
Cl1—C7	1.728 (3)	Cl2—C26	1.723 (3)
O1—C6	1.240 (4)	O3—C27	1.230 (4)
O2—C15	1.365 (4)	N6—C25	1.345 (4)
O2—C18	1.431 (4)	N6—C28	1.451 (4)
C8—N3	1.350 (4)	N6—H6	0.8800
C8—C7	1.378 (4)	N4—N5	1.351 (4)
C8—C9	1.440 (4)	N4—C27	1.383 (4)
C12—C13	1.392 (5)	N4—C23	1.470 (4)
C12—C17	1.393 (4)	N5—C24	1.293 (4)
C12—C11	1.501 (4)	O4—C33	1.373 (4)
N3—C10	1.459 (4)	O4—C36	1.423 (5)
N3—H3	0.8800	C35—C34	1.381 (5)
N2—C9	1.297 (4)	C35—C30	1.391 (4)
N2—N1	1.344 (3)	C35—H35	0.9500
N1—C6	1.388 (4)	C26—C25	1.376 (4)
N1—C5	1.470 (4)	C26—C27	1.436 (4)
C6—C7	1.425 (4)	C25—C24	1.446 (4)
C1—C2	1.517 (4)	C20—C19	1.518 (4)
C1—H1A	0.9900	C20—C21	1.518 (5)
C1—H1B	0.9900	C20—H20A	0.9900
C15—C16	1.388 (4)	C20—H20B	0.9900
C15—C14	1.394 (4)	C24—H24	0.9500
C9—H9	0.9500	C34—C33	1.387 (5)
C17—C16	1.392 (4)	C34—H34	0.9500
C17—H17	0.9500	C28—C29	1.535 (4)
C10—C11	1.530 (4)	C28—H28A	0.9900
C10—H10A	0.9900	C28—H28B	0.9900
C10—H10B	0.9900	C32—C31	1.386 (5)
C16—H16	0.9500	C32—C33	1.396 (5)
C14—C13	1.381 (5)	C32—H32	0.9500
C14—H14	0.9500	C31—C30	1.390 (5)
C4—C5	1.518 (5)	C31—H31	0.9500
C4—C3	1.532 (5)	C29—C30	1.505 (4)
C4—H4A	0.9900	C29—H29A	0.9900
C4—H4B	0.9900	C29—H29B	0.9900
C13—H13	0.9500	C23—C22	1.517 (5)
C2—C3	1.515 (4)	C23—H23A	0.9900
C2—H2A	0.9900	C23—H23B	0.9900
C2—H2B	0.9900	C22—C21	1.523 (4)
C3—H3A	0.9900	C22—H22A	0.9900
C3—H3B	0.9900	C22—H22B	0.9900
C11—H11A	0.9900	C21—H21A	0.9900
C11—H11B	0.9900	C21—H21B	0.9900
C18—H18A	0.9800	C36—H36A	0.9800
C18—H18B	0.9800	C36—H36B	0.9800
C18—H18C	0.9800	C36—H36C	0.9800
C5—H5A	0.9900	C19—H19A	0.9900
C5—H5B	0.9900	C19—H19B	0.9900
			
C15—O2—C18	117.8 (2)	C25—N6—C28	123.6 (3)
N3—C8—C7	124.2 (3)	C25—N6—H6	118.2
N3—C8—C9	121.2 (3)	C28—N6—H6	118.2
C7—C8—C9	114.6 (3)	N5—N4—C27	125.5 (3)
C13—C12—C17	117.5 (3)	N5—N4—C23	114.9 (3)
C13—C12—C11	120.6 (3)	C27—N4—C23	119.6 (3)
C17—C12—C11	121.9 (3)	C24—N5—N4	117.8 (3)
C8—N3—C10	122.4 (3)	C33—O4—C36	116.8 (3)
C8—N3—H3	118.8	C34—C35—C30	121.2 (3)
C10—N3—H3	118.8	C34—C35—H35	119.4
C9—N2—N1	118.1 (3)	C30—C35—H35	119.4
N2—N1—C6	125.4 (2)	C25—C26—C27	122.8 (3)
N2—N1—C5	114.3 (3)	C25—C26—Cl2	120.1 (2)
C6—N1—C5	120.3 (2)	C27—C26—Cl2	117.1 (2)
O1—C6—N1	120.7 (3)	N6—C25—C26	124.2 (3)
O1—C6—C7	125.1 (3)	N6—C25—C24	121.2 (3)
N1—C6—C7	114.3 (3)	C26—C25—C24	114.6 (3)
C2—C1—Br1	111.3 (2)	O3—C27—N4	120.8 (3)
C2—C1—H1A	109.4	O3—C27—C26	124.9 (3)
Br1—C1—H1A	109.4	N4—C27—C26	114.3 (3)
C2—C1—H1B	109.4	C19—C20—C21	109.5 (3)
Br1—C1—H1B	109.4	C19—C20—H20A	109.8
H1A—C1—H1B	108.0	C21—C20—H20A	109.8
O2—C15—C16	125.3 (3)	C19—C20—H20B	109.8
O2—C15—C14	115.1 (3)	C21—C20—H20B	109.8
C16—C15—C14	119.5 (3)	H20A—C20—H20B	108.2
N2—C9—C8	124.6 (3)	N5—C24—C25	125.0 (3)
N2—C9—H9	117.7	N5—C24—H24	117.5
C8—C9—H9	117.7	C25—C24—H24	117.5
C16—C17—C12	121.9 (3)	C35—C34—C33	120.3 (3)
C16—C17—H17	119.0	C35—C34—H34	119.9
C12—C17—H17	119.0	C33—C34—H34	119.9
N3—C10—C11	112.6 (3)	N6—C28—C29	112.3 (3)
N3—C10—H10A	109.1	N6—C28—H28A	109.1
C11—C10—H10A	109.1	C29—C28—H28A	109.1
N3—C10—H10B	109.1	N6—C28—H28B	109.1
C11—C10—H10B	109.1	C29—C28—H28B	109.1
H10A—C10—H10B	107.8	H28A—C28—H28B	107.9
C15—C16—C17	119.3 (3)	C31—C32—C33	119.1 (3)
C15—C16—H16	120.3	C31—C32—H32	120.5
C17—C16—H16	120.3	C33—C32—H32	120.5
C8—C7—C6	123.1 (3)	C32—C31—C30	122.0 (3)
C8—C7—Cl1	120.1 (2)	C32—C31—H31	119.0
C6—C7—Cl1	116.8 (2)	C30—C31—H31	119.0
C13—C14—C15	120.1 (3)	C30—C29—C28	113.9 (3)
C13—C14—H14	119.9	C30—C29—H29A	108.8
C15—C14—H14	119.9	C28—C29—H29A	108.8
C5—C4—C3	113.0 (3)	C30—C29—H29B	108.8
C5—C4—H4A	109.0	C28—C29—H29B	108.8
C3—C4—H4A	109.0	H29A—C29—H29B	107.7
C5—C4—H4B	109.0	C31—C30—C35	117.8 (3)
C3—C4—H4B	109.0	C31—C30—C29	120.5 (3)
H4A—C4—H4B	107.8	C35—C30—C29	121.7 (3)
C14—C13—C12	121.6 (3)	O4—C33—C34	116.5 (3)
C14—C13—H13	119.2	O4—C33—C32	123.8 (3)
C12—C13—H13	119.2	C34—C33—C32	119.7 (3)
C3—C2—C1	113.0 (3)	N4—C23—C22	113.1 (2)
C3—C2—H2A	109.0	N4—C23—H23A	109.0
C1—C2—H2A	109.0	C22—C23—H23A	109.0
C3—C2—H2B	109.0	N4—C23—H23B	109.0
C1—C2—H2B	109.0	C22—C23—H23B	109.0
H2A—C2—H2B	107.8	H23A—C23—H23B	107.8
C2—C3—C4	114.1 (3)	C23—C22—C21	110.6 (3)
C2—C3—H3A	108.7	C23—C22—H22A	109.5
C4—C3—H3A	108.7	C21—C22—H22A	109.5
C2—C3—H3B	108.7	C23—C22—H22B	109.5
C4—C3—H3B	108.7	C21—C22—H22B	109.5
H3A—C3—H3B	107.6	H22A—C22—H22B	108.1
C12—C11—C10	113.7 (3)	C20—C21—C22	113.7 (3)
C12—C11—H11A	108.8	C20—C21—H21A	108.8
C10—C11—H11A	108.8	C22—C21—H21A	108.8
C12—C11—H11B	108.8	C20—C21—H21B	108.8
C10—C11—H11B	108.8	C22—C21—H21B	108.8
H11A—C11—H11B	107.7	H21A—C21—H21B	107.7
O2—C18—H18A	109.5	O4—C36—H36A	109.5
O2—C18—H18B	109.5	O4—C36—H36B	109.5
H18A—C18—H18B	109.5	H36A—C36—H36B	109.5
O2—C18—H18C	109.5	O4—C36—H36C	109.5
H18A—C18—H18C	109.5	H36A—C36—H36C	109.5
H18B—C18—H18C	109.5	H36B—C36—H36C	109.5
N1—C5—C4	111.2 (3)	C20—C19—Br2	112.5 (2)
N1—C5—H5A	109.4	C20—C19—H19A	109.1
C4—C5—H5A	109.4	Br2—C19—H19A	109.1
N1—C5—H5B	109.4	C20—C19—H19B	109.1
C4—C5—H5B	109.4	Br2—C19—H19B	109.1
H5A—C5—H5B	108.0	H19A—C19—H19B	107.8
			
C7—C8—N3—C10	169.7 (3)	C27—N4—N5—C24	−2.6 (4)
C9—C8—N3—C10	−11.8 (4)	C23—N4—N5—C24	178.0 (3)
C9—N2—N1—C6	−0.8 (4)	C28—N6—C25—C26	170.9 (3)
C9—N2—N1—C5	−177.0 (3)	C28—N6—C25—C24	−11.1 (4)
N2—N1—C6—O1	−177.7 (3)	C27—C26—C25—N6	178.7 (3)
C5—N1—C6—O1	−1.7 (4)	Cl2—C26—C25—N6	−3.2 (4)
N2—N1—C6—C7	1.5 (4)	C27—C26—C25—C24	0.5 (4)
C5—N1—C6—C7	177.5 (3)	Cl2—C26—C25—C24	178.6 (2)
C18—O2—C15—C16	12.9 (4)	N5—N4—C27—O3	−176.9 (3)
C18—O2—C15—C14	−167.0 (3)	C23—N4—C27—O3	2.6 (4)
N1—N2—C9—C8	0.0 (5)	N5—N4—C27—C26	3.9 (4)
N3—C8—C9—N2	−178.7 (3)	C23—N4—C27—C26	−176.7 (3)
C7—C8—C9—N2	0.0 (5)	C25—C26—C27—O3	178.1 (3)
C13—C12—C17—C16	−1.7 (4)	Cl2—C26—C27—O3	−0.1 (4)
C11—C12—C17—C16	178.4 (3)	C25—C26—C27—N4	−2.7 (4)
C8—N3—C10—C11	−74.9 (4)	Cl2—C26—C27—N4	179.2 (2)
O2—C15—C16—C17	179.4 (3)	N4—N5—C24—C25	−0.2 (5)
C14—C15—C16—C17	−0.7 (4)	N6—C25—C24—N5	−177.2 (3)
C12—C17—C16—C15	1.4 (5)	C26—C25—C24—N5	1.1 (5)
N3—C8—C7—C6	179.4 (3)	C30—C35—C34—C33	0.7 (5)
C9—C8—C7—C6	0.8 (4)	C25—N6—C28—C29	−75.2 (4)
N3—C8—C7—Cl1	−2.5 (4)	C33—C32—C31—C30	−0.9 (5)
C9—C8—C7—Cl1	178.9 (2)	N6—C28—C29—C30	160.2 (3)
O1—C6—C7—C8	177.7 (3)	C32—C31—C30—C35	0.4 (5)
N1—C6—C7—C8	−1.5 (4)	C32—C31—C30—C29	179.6 (3)
O1—C6—C7—Cl1	−0.4 (4)	C34—C35—C30—C31	−0.3 (5)
N1—C6—C7—Cl1	−179.6 (2)	C34—C35—C30—C29	−179.5 (3)
O2—C15—C14—C13	−179.8 (3)	C28—C29—C30—C31	−114.7 (3)
C16—C15—C14—C13	0.3 (5)	C28—C29—C30—C35	64.4 (4)
C15—C14—C13—C12	−0.6 (5)	C36—O4—C33—C34	178.4 (3)
C17—C12—C13—C14	1.3 (5)	C36—O4—C33—C32	−1.8 (5)
C11—C12—C13—C14	−178.8 (3)	C35—C34—C33—O4	178.7 (3)
Br1—C1—C2—C3	69.2 (3)	C35—C34—C33—C32	−1.1 (5)
C1—C2—C3—C4	−177.7 (3)	C31—C32—C33—O4	−178.6 (3)
C5—C4—C3—C2	−60.4 (4)	C31—C32—C33—C34	1.2 (5)
C13—C12—C11—C10	−101.8 (3)	N5—N4—C23—C22	91.8 (3)
C17—C12—C11—C10	78.1 (4)	C27—N4—C23—C22	−87.7 (4)
N3—C10—C11—C12	156.1 (3)	N4—C23—C22—C21	−175.9 (3)
N2—N1—C5—C4	75.2 (3)	C19—C20—C21—C22	−169.8 (3)
C6—N1—C5—C4	−101.2 (3)	C23—C22—C21—C20	175.8 (3)
C3—C4—C5—N1	−169.8 (3)	C21—C20—C19—Br2	175.9 (2)

### Hydrogen-bond geometry (Å, °)

**Table:**

*D*—H···*A*	*D*—H	H···*A*	*D*···*A*	*D*—H···*A*
N3—H3···O3^i^	0.88	2.11	2.796 (3)	135.
N6—H6···O1^ii^	0.88	2.06	2.815 (3)	143.

Symmetry codes: (i) *x*−1, *y*+1, *z*−1; (ii) *x*+1, *y*, *z*+1.
